# Supplementary material for: Pb2+ biosorption from aqueous solutions by live and dead biosorbents of the hydrocarbon-degrading strain Rhodococcus sp. HX-2
Source: PLoS One. 2020 Jan 29;15(1):e0226557. doi: 10.1371/journal.pone.0226557 (PMC6988972; doi:10.1371/journal.pone.0226557)
Supplement: S1 Table — (PDF) [file pone.0226557.s001.pdf]

S1 Table. Variables and levels for Box-Behnken design.

Variables	Symbols	Coded levels		
		Low factorial (-1)	Center point (0)	High factorial (+1)
Biosorbent dose (g)	X_1	0.5	0.75	1
pH	X_2	4	5	6
Temperature (°C)	X_3	15	20	25
Contact time (min)	X_4	5	7.5	10
