# Supplementary material for: Pb2+ biosorption from aqueous solutions by live and dead biosorbents of the hydrocarbon-degrading strain Rhodococcus sp. HX-2
Source: PLoS One. 2020 Jan 29;15(1):e0226557. doi: 10.1371/journal.pone.0226557 (PMC6988972; doi:10.1371/journal.pone.0226557)
Supplement: S2 Table — (PDF) [file pone.0226557.s002.pdf]

S2 Table. Box Behnken design matrix and response values.

Run	X_1/Biosorbent dose (g)	X_2/pH	X_3/Temperature (°C)	X_4/Contact time (min)	Y/Biosorption capacity (mg g^{-1})
1	0.50	6.00	20.00	10.00	124.713
2	0.50	5.00	25.00	10.00	120.014
3	0.50	5.00	20.00	5.00	183.032
4	1.00	5.00	25.00	7.50	95.7281
5	1.00	4.00	20.00	7.50	94.983
6	0.50	5.00	25.00	5.00	122.582
7	1.00	5.00	15.00	7.50	94.8548
8	0.50	5.00	15.00	10.00	119.439
9	0.50	5.00	20.00	7.50	128.323
10	0.50	5.00	20.00	7.50	125.267
11	0.50	4.00	20.00	10.00	99.7334
12	1.00	5.00	20.00	10.00	95.2613
13	0.50	4.00	15.00	7.50	104.787
14	0.75	5.00	25.00	7.50	179.331
15	0.50	5.00	20.00	7.50	120.24
16	0.75	4.00	20.00	7.50	191.855
17	0.50	5.00	15.00	5.00	123.719
18	0.50	6.00	20.00	5.00	122.6
19	0.75	5.00	15.00	7.50	185.415

20	1.00	6.00	20.00	7.50	94.9313
21	0.50	5.00	20.00	7.50	126.919
22	0.50	6.00	25.00	7.50	127.728
23	0.75	5.00	20.00	10.00	188.195
24	0.50	4.00	20.00	5.00	126.934
25	0.50	6.00	15.00	7.50	123.664
26	0.50	4.00	25.00	7.50	126.286
27	1.00	5.00	20.00	5.00	95.8284
28	0.75	6.00	20.00	7.50	179.779
29	0.50	5.00	20.00	7.50	121.777
