# Supplementary material for: Pb2+ biosorption from aqueous solutions by live and dead biosorbents of the hydrocarbon-degrading strain Rhodococcus sp. HX-2
Source: PLoS One. 2020 Jan 29;15(1):e0226557. doi: 10.1371/journal.pone.0226557 (PMC6988972; doi:10.1371/journal.pone.0226557)
Supplement: S3 Table — (PDF) [file pone.0226557.s003.pdf]

S3 Table. Analysis of variance for the response surface quadratic model.

Source	Sum of Squares	Degree of freedom	Mean Square	*F* Value	Probability (Prob>*F*)
Model	24041.20	14	1717.23	63.19	< 0.0001
X_1 −Biosorbent dose	3170.89	1	3170.89	116.69	< 0.0001
X_2 −pH	2.13	1	2.13	0.08	0.7837
X_3 −Temperature	3.36	1	3.36	0.12	0.7303
X_4 −Contacttime	20.13	1	20.13	0.74	0.404
X_1X_2	101.07	1	101.07	3.72	0.0743
X_1X_3	28.94	1	28.94	1.07	0.3196
X_1X_4	36.71	1	36.71	1.35	0.2645
X_2X_3	76.00	1	76.00	2.80	0.1166
X_2X_4	214.82	1	214.82	7.91	0.0139
X_3X_4	0.73	1	0.73	0.03	0.8719
${X_1}^2$	21672.98	1	21672.98	797.55	< 0.0001
${X_2}^2$	55.11	1	55.11	2.03	0.1763
${X_3}^2$	31.85	1	31.85	1.17	0.2973
${X_4}^2$	15.07	1	15.07	0.55	0.4688
Residual	380.44	14	27.17		
Lack of Fit	333.82	10	33.38	2.86	0.1611
Pure Error	46.62	4	11.66		
Cor Total	24421.64	28			

R^2= 0.9844*;* *AdjR*^{2}= 0.9688; C.V.= 0.41; Adeq Precision: 25.27.
