# Supplementary material for: Pb2+ biosorption from aqueous solutions by live and dead biosorbents of the hydrocarbon-degrading strain Rhodococcus sp. HX-2
Source: PLoS One. 2020 Jan 29;15(1):e0226557. doi: 10.1371/journal.pone.0226557 (PMC6988972; doi:10.1371/journal.pone.0226557)
Supplement: S4 Table — (PDF) [file pone.0226557.s004.pdf]

S4 Table. The predicted and experimental value of response under optimum conditions

	Biosorbent dose (g)	pH	Temperature (℃)	Contact time （h）	Biosorption capacity (mg g^{-1})
Optimum conditions	0.83	4	24.41	3	180.957
Experimental conditions	0.83	4	24	3	179.541
