# Supplementary material for: Pb2+ biosorption from aqueous solutions by live and dead biosorbents of the hydrocarbon-degrading strain Rhodococcus sp. HX-2
Source: PLoS One. 2020 Jan 29;15(1):e0226557. doi: 10.1371/journal.pone.0226557 (PMC6988972; doi:10.1371/journal.pone.0226557)
Supplement: S5 Table — (PDF) [file pone.0226557.s005.pdf]

S5 Table. Langmuir adsorption isotherms and Freundlich adsorption isotherms for Pb^{2+} using live and dead biosorbents

Strain	Metal ions	Langmuir constants			Freundlich constants		
		b (L mg^{-1})	q_{max} (mg g^{-1})	R^2	1/n	*K*	R^2
Live	Pb	0.091	89.63	0.8205	0.7747	1.2999	0.9449
Dead	Pb	0.018	187.52	0.8966	0.3576	19.3108	0.9633
