# Supplementary material for: Pb2+ biosorption from aqueous solutions by live and dead biosorbents of the hydrocarbon-degrading strain Rhodococcus sp. HX-2
Source: PLoS One. 2020 Jan 29;15(1):e0226557. doi: 10.1371/journal.pone.0226557 (PMC6988972; doi:10.1371/journal.pone.0226557)
Supplement: S6 Table — (PDF) [file pone.0226557.s006.pdf]

S6 Table. Pseudo-first-order adsorption kinetic constants of the live and dead biosorbents.

Metal ions (II)	Biosorbent	C_0(mg L^{-1})	q_e,the.(mg g^{-1})	q_e,exp.(mg g^{-1})	Presudo-first-order model		
					q_e,cal. (mg g^{-1})	K_1 (×$10^{-3}$$min^{-1}$)	R^2
Pb	Live	123.0185	162.5795	66.9218	64.7800	0.0090	0.9711
	Dead	123.0185	162.5795	112.4182	93.0400	0.0099	0.9589
