# Supplementary material for: Pb2+ biosorption from aqueous solutions by live and dead biosorbents of the hydrocarbon-degrading strain Rhodococcus sp. HX-2
Source: PLoS One. 2020 Jan 29;15(1):e0226557. doi: 10.1371/journal.pone.0226557 (PMC6988972; doi:10.1371/journal.pone.0226557)
Supplement: S7 Table — (PDF) [file pone.0226557.s007.pdf]

S7 Table. Pseudo-second-order adsorption kinetic constants of the live and dead biosorbents.

Metal ions (II)	biosorbent	C_0 (mg L^{-1})	q_e,the.(mg g^{-1})	q_e,exp.(mgg^{-1})	Presudo-second-order model		
					q_e,cal.(mg g^{-1})	K_2(g mg^{-1} min^{-1})	R^2
Pb	Live	123.0185	162.5795	72.0221	71.9424	3.8195×10^{-4}	0.9958
	Dead	123.0185	162.5795	119.3032	119.0476	3.8665×10^{-4}	0.9970
