# Supplementary material for: Pb2+ biosorption from aqueous solutions by live and dead biosorbents of the hydrocarbon-degrading strain Rhodococcus sp. HX-2
Source: PLoS One. 2020 Jan 29;15(1):e0226557. doi: 10.1371/journal.pone.0226557 (PMC6988972; doi:10.1371/journal.pone.0226557)
Supplement: S8 Table — (PDF) [file pone.0226557.s008.pdf]

S8 Table. Intra-particle diffusion model constants and correlation coefficients for adsorption of metal ions on the live and dead biosorbents.

Metal Ions(Pb^{2+})	C_0 (mg L^{-1})	Intra-particle diffusion model								
		K_{d1} (mg g^{-1} min $^{1/2}$)	K_{d2} (mg g^{-1} min $^{1/2}$)	K_{d3} (mg g^{-1} min $^{1/2}$)	C_1	C_2	C_3	$(R_1)^2$	$(R_2)^2$	$(R_3)^2$
Live	200	31.0948	1.9255	0.2487	-3.5924	21.8675	39.6182	0.9613	0.9533	0.7731
Dead	200	44.7160	4.0943	1.0985	-3.1426	16.6212	30.6531	0.9709	0.9521	0.9339
