# Supplementary material for: Pb2+ biosorption from aqueous solutions by live and dead biosorbents of the hydrocarbon-degrading strain Rhodococcus sp. HX-2
Source: PLoS One. 2020 Jan 29;15(1):e0226557. doi: 10.1371/journal.pone.0226557 (PMC6988972; doi:10.1371/journal.pone.0226557)
Supplement: S9 Table — (PDF) [file pone.0226557.s009.pdf]

S9 Table. EDX (SEM) analysis for natural biosorbent

Element	Line type	Apparent concentration	K value	Wt (%)	Wt (%) Sigma	Atomic percentage
C	K line	48.80	0.48796	85.03	2.05	89.93
N	K line	3.48	0.00620	7.50	2.23	6.80
Na	K line	0.29	0.00122	0.20	0.04	0.11
Mg	K line	1.80	0.01195	1.58	0.06	0.83
P	K line	8.55	0.04780	5.38	0.15	2.21
S	K line	0.32	0.00278	0.31	0.04	0.12
Gross:				100		100
