# Supplementary material for: Pb2+ biosorption from aqueous solutions by live and dead biosorbents of the hydrocarbon-degrading strain Rhodococcus sp. HX-2
Source: PLoS One. 2020 Jan 29;15(1):e0226557. doi: 10.1371/journal.pone.0226557 (PMC6988972; doi:10.1371/journal.pone.0226557)
Supplement: S10 Table — (PDF) [file pone.0226557.s010.pdf]

**S10 Table.** EDX (SEM) analysis for $Pb^{2+}$ loaded dead biosorbent

| Element | Line type | Apparent concentration | K value | Wt (%) | Wt (%) Sigma | Atomic percentage |
|---|---|---|---|---|---|---|
| C | K line | 7.95 | 0.07946 | 35.66 | 0.60 | 73.79 |
| N | K line | 1.08 | 0.00191 | 1.73 | 0.82 | 3.07 |
| O | K line | 4.04 | 0.01360 | 8.55 | 0.29 | 13.29 |
| Na | K line | 0.21 | 0.00088 | 0.27 | 0.06 | 0.29 |
| P | K line | 5.03 | 0.02813 | 4.54 | 0.12 | 3.65 |
| Pb | M line | 26.53 | 0.24684 | 49.23 | 0.67 | 5.91 |
| Gross: | | | | 100 | | 100 |
