# Supplementary material for: Pb2+ biosorption from aqueous solutions by live and dead biosorbents of the hydrocarbon-degrading strain Rhodococcus sp. HX-2
Source: PLoS One. 2020 Jan 29;15(1):e0226557. doi: 10.1371/journal.pone.0226557 (PMC6988972; doi:10.1371/journal.pone.0226557)
Supplement: S11 Table — (PDF) [file pone.0226557.s011.pdf]

S11 Table. EDX (SEM) analysis for Pb^{2+} loaded live biosorbent

Element	Line type	Apparent concentration	K value	Wt (%)	Wt (%) Sigma	Atomic percentage
C	K line	23.48	0.23477	72.67	0.85	85.09
N	K line	0.00	0.00000	0.00	0.00	0.00
O	K line	5.04	0.01696	14.12	0.61	12.41
Na	K line	0.01	0.00006	0.02	0.09	0.01
P	K line	3.87	0.02163	4.11	0.19	1.86
Pb	M line	4.32	0.04023	9.08	0.76	0.62
Gross:				100		100
