# Supplementary material for: Pb2+ biosorption from aqueous solutions by live and dead biosorbents of the hydrocarbon-degrading strain Rhodococcus sp. HX-2
Source: PLoS One. 2020 Jan 29;15(1):e0226557. doi: 10.1371/journal.pone.0226557 (PMC6988972; doi:10.1371/journal.pone.0226557)
Supplement: S12 Table — (PDF) [file pone.0226557.s012.pdf]

S12 Table. EDX (TEM) analysis for native biosorbent

Element	Family	Atomic Fraction (%)	Atomic Error (%)	Mass Fraction (%)	Mass Error (%)	Fit error (%)
C	K	77.85	3.85	73.34	2.80	0.07
N	K	9.27	1.92	9.98	2.04	1.68
O	K	12.68	2.61	16.20	3.30	0.39
S	K	0.20	0.04	0.47	0.09	2.46
