# Supplementary material for: Pb2+ biosorption from aqueous solutions by live and dead biosorbents of the hydrocarbon-degrading strain Rhodococcus sp. HX-2
Source: PLoS One. 2020 Jan 29;15(1):e0226557. doi: 10.1371/journal.pone.0226557 (PMC6988972; doi:10.1371/journal.pone.0226557)
Supplement: S13 Table — (PDF) [file pone.0226557.s013.pdf]

S13 Table. EDX (TEM)analysis for Pb^{2+} loaded dead biosorbent

Element	Family	Atomic Fraction (%)	Atomic Error (%)	Mass Fraction (%)	Mass Error (%)	Fit error (%)
C	K	44.84	4.87	7.25	0.70	0.95
N	K	0.00	0.04	0.00	0.01	0.00
O	K	14.48	3.30	3.18	0.71	1.69
S	K	0.00	0.13	0.00	0.05	0.00
Pb	L	36.50	5.60	88.03	12.76	0.10
