# Supplementary material for: Pb2+ biosorption from aqueous solutions by live and dead biosorbents of the hydrocarbon-degrading strain Rhodococcus sp. HX-2
Source: PLoS One. 2020 Jan 29;15(1):e0226557. doi: 10.1371/journal.pone.0226557 (PMC6988972; doi:10.1371/journal.pone.0226557)
Supplement: S14 Table — (PDF) [file pone.0226557.s014.pdf]

S14 Table. EDX (TEM) for Pb^{2+} loaded live biosorbent

Element	Family	Atomic Fraction (%)	Atomic Error (%)	Mass Fraction (%)	Mass Error (%)	Fit error (%)
C	K	80.93	3.25	75.74	2.30	0.33
N	K	10.24	2.09	10.94	2.21	0.68
O	K	8.06	1.65	10.23	2.07	1.24
S	K	0.12	0.02	0.29	0.06	5.36
Pb	L	0.12	0.01	1.64	0.19	1.89
