# Supplementary figures and images for: Pb2+ biosorption from aqueous solutions by live and dead biosorbents of the hydrocarbon-degrading strain Rhodococcus sp. HX-2
Source: PLoS One. 2020 Jan 29;15(1):e0226557. doi: 10.1371/journal.pone.0226557 (PMC6988972; doi:10.1371/journal.pone.0226557)

Predicted

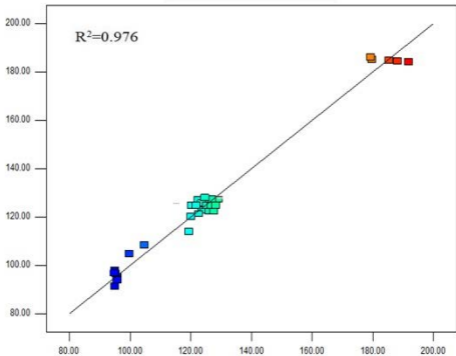

Actual

Supplement: S1 Fig — (PDF) [file pone.0226557.s015.pdf]

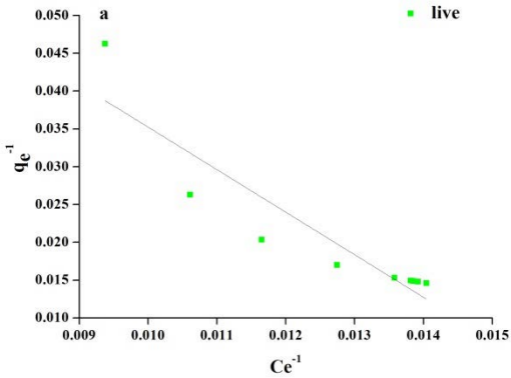

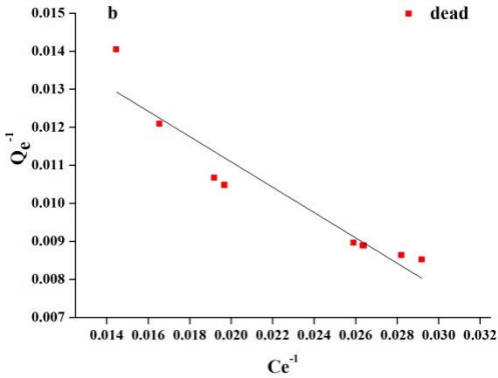

Supplement: S3 Fig — Langmuir adsorption isotherms for Pb2+ using live (a) and dead biosorbents (b). (PDF) [file pone.0226557.s017.pdf]

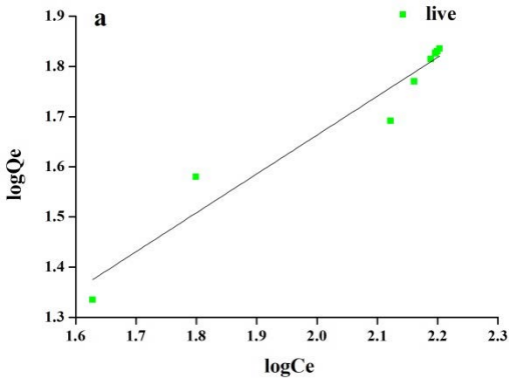

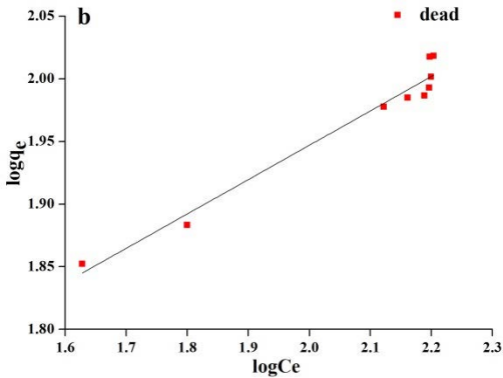

Supplement: S4 Fig — Freundlich adsorption isotherms for Pb2+ using live (a) and dead biosorbents (b). (PDF) [file pone.0226557.s018.pdf]

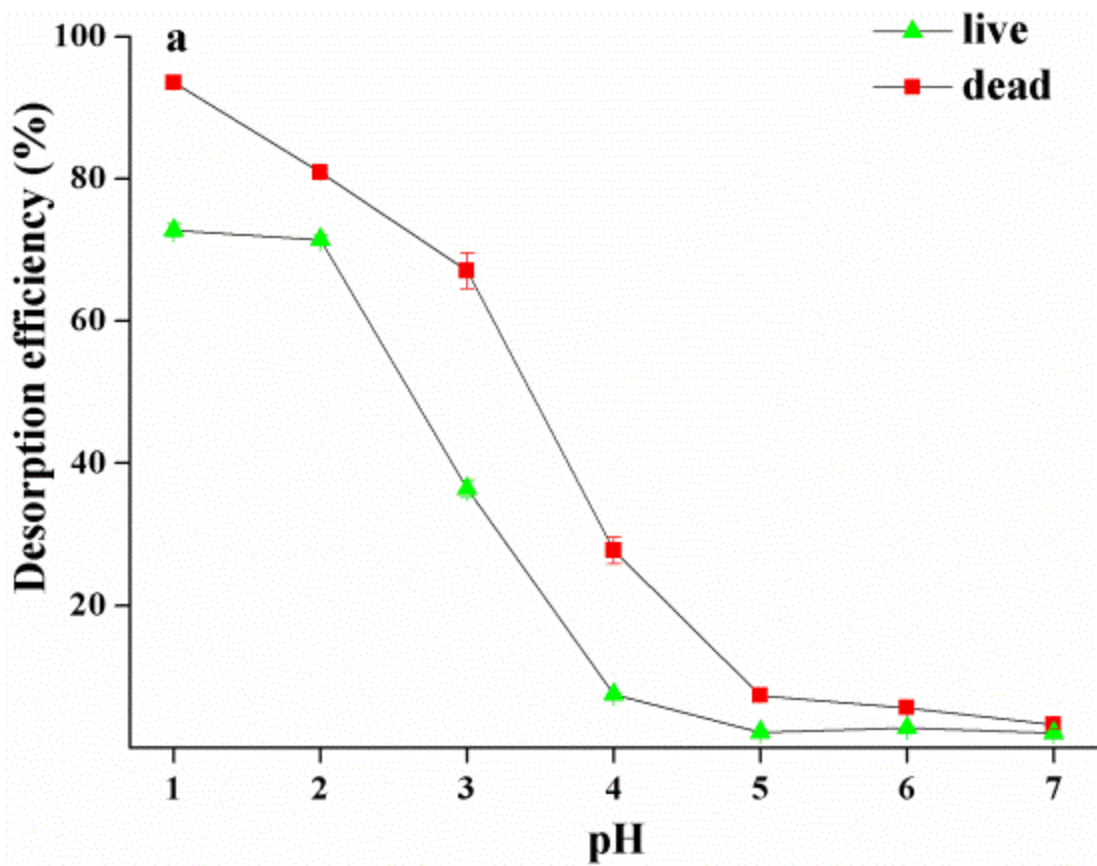

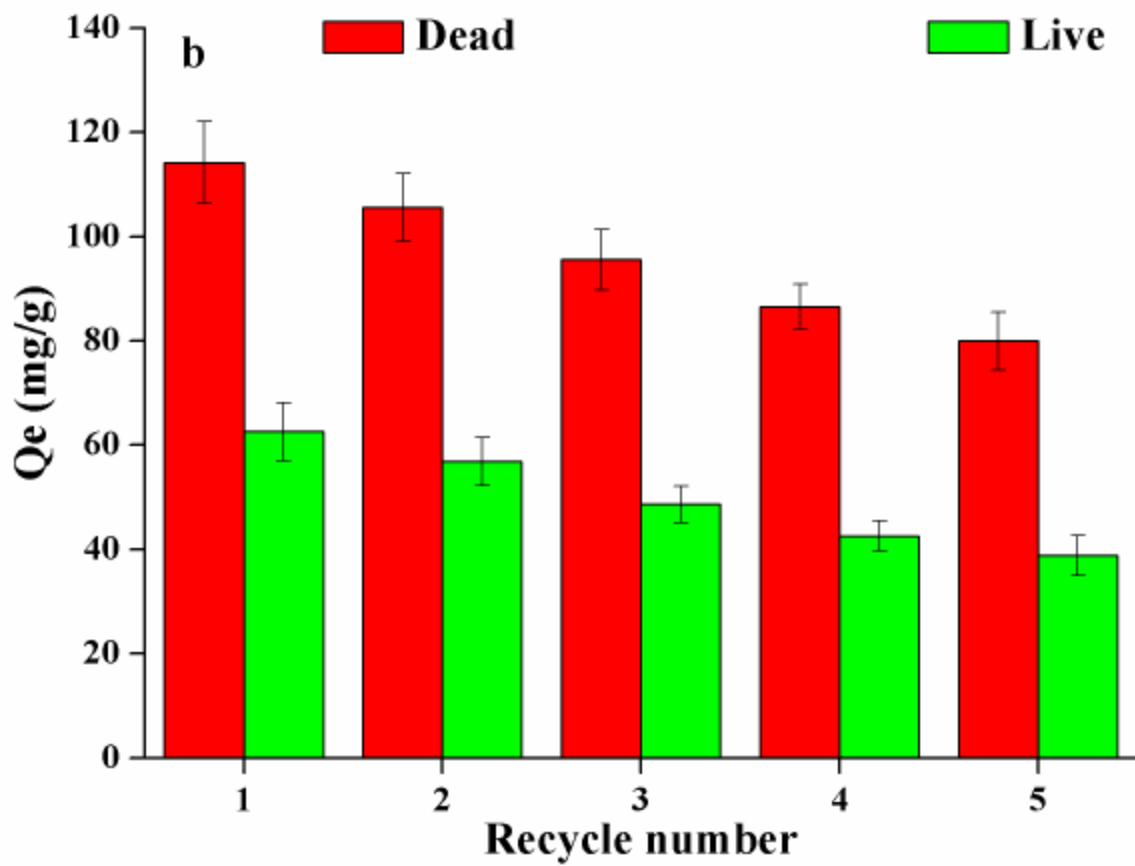

Supplement: S5 Fig — Effect of pH on desorption efficiency of Pb2+ from live and dead biosorbents (a) and the recycling experiments (b). (PDF) [file pone.0226557.s019.pdf]

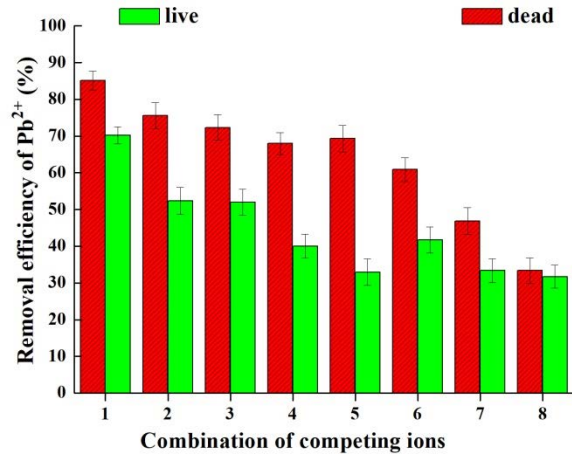

Supplement: S6 Fig — (PDF) [file pone.0226557.s020.pdf]
