# Supplementary material for: Pb2+ biosorption from aqueous solutions by live and dead biosorbents of the hydrocarbon-degrading strain Rhodococcus sp. HX-2
Source: PLoS One. 2020 Jan 29;15(1):e0226557. doi: 10.1371/journal.pone.0226557 (PMC6988972; doi:10.1371/journal.pone.0226557)
Supplement: S2 Fig — (PDF) [file pone.0226557.s016.pdf]

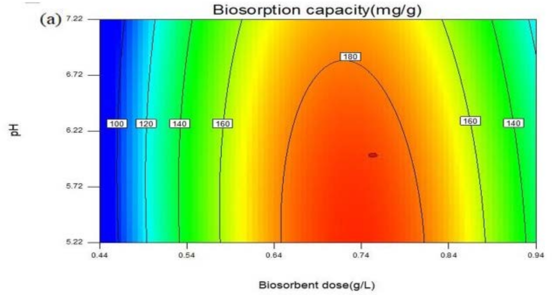
(a)
Biosorption capacity(mg/g)
pH
7.22
6.72
6.22
5.72
5.22
0.44
0.54
0.64
0.74
0.84
0.94
100
120
140
160
180
160
140
Biosorbent dose(g/L)

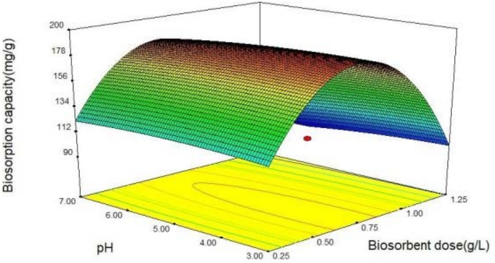

Biosorption capacity(mg/g)
200
178
156
134
112
90
pH
7.00
6.00
5.00
4.00
3.00
Biosorbent dose(g/L)
0.25
0.50
0.75
1.00
1.25

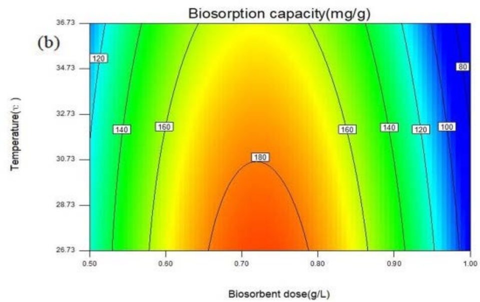
Biosorption capacity(mg/g)
(b)
120
140
160
180
160
140
120
100
80
Temperature(℃)
36.73
34.73
32.73
30.73
28.73
26.73
0.50
0.60
0.70
0.80
0.90
1.00
Biosorbent dose(g/L)

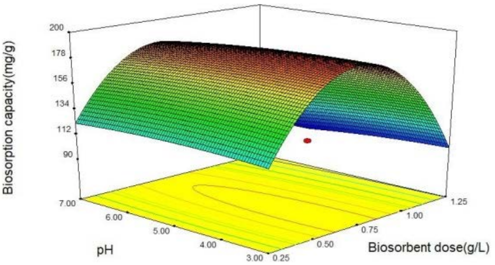

Biosorption capacity(mg/g)
200
178
156
134
112
90
7.00
6.00
5.00
4.00
3.00
pH
0.25
0.50
0.75
1.00
1.25
Biosorbent dose(g/L)

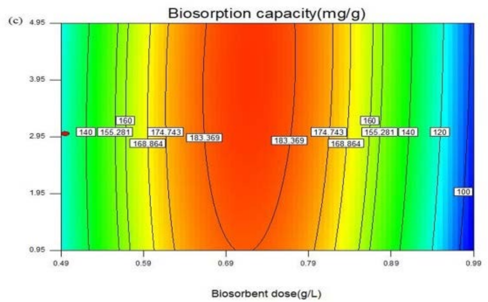

(c)
Biosorption capacity(mg/g)
Time(h)
4.95
3.95
2.95
1.95
0.95
160
140
155.281
174.743
168.864
183.369
183.369
174.743
168.864
160
155.281
140
120
100
0.49
0.59
0.69
0.79
0.89
0.99
Biosorbent dose(g/L)

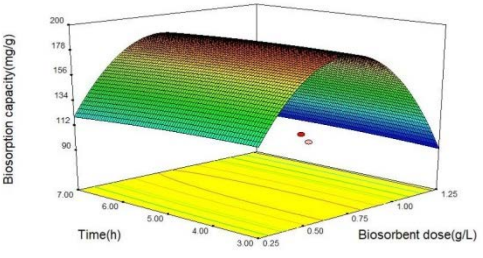

Biosorption capacity(mg/g)
200
178
156
134
112
90
Time(h)
7.00
6.00
5.00
4.00
3.00
Biosorbent dose(g/L)
0.25
0.50
0.75
1.00
1.25

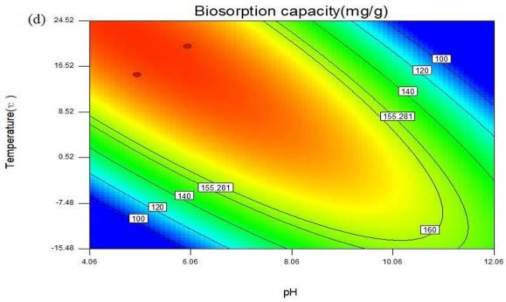
(d)
Biosorption capacity(mg/g)
Temperature(℃)
pH
24.52
16.52
8.52
0.52
-7.48
-15.48
4.06
6.06
8.06
10.06
12.06
100
120
140
155.281
160

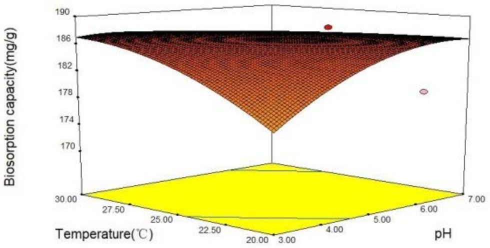

190
186
182
178
174
170
Biosorption capacity(mg/g)
30.00
27.50
25.00
22.50
20.00
Temperature(℃)
3.00
4.00
5.00
6.00
7.00
pH

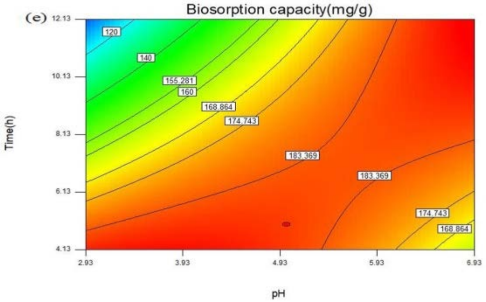
(e)
Biosorption capacity(mg/g)
Time(h)
12.13
10.13
8.13
6.13
4.13
2.93
3.93
4.93
5.93
6.93
pH
120
140
155.281
160
168.864
174.743
183.369
183.369
174.743
168.864

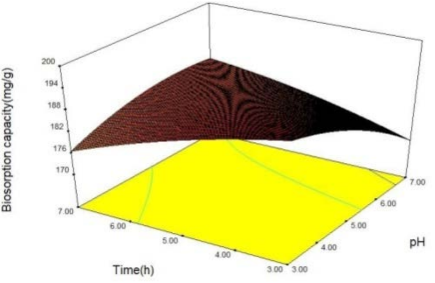

Biosorption capacity(mg/g)
200
194
188
182
176
170
7.00
6.00
5.00
4.00
3.00
Time(h)
3.00
4.00
5.00
6.00
7.00
pH

(f)

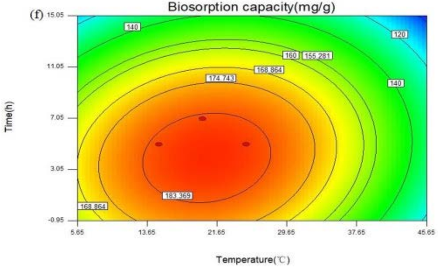
Biosorption capacity(mg/g)
15.05
11.05
7.05
3.05
-0.95
Time(h)
5.65
13.65
21.65
29.65
37.65
45.65
Temperature(℃)
140
120
160
155.281
168.864
174.743
140
183.369
168.864

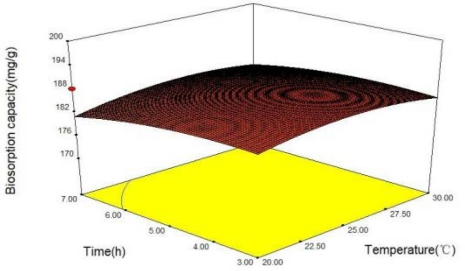

Biosorption capacity(mg/g)
200
194
188
182
176
170
7.00
6.00
5.00
4.00
3.00
Time(h)
20.00
22.50
25.00
27.50
30.00
Temperature(℃)
