# Supplementary material for: Pb2+ biosorption from aqueous solutions by live and dead biosorbents of the hydrocarbon-degrading strain Rhodococcus sp. HX-2
Source: PLoS One. 2020 Jan 29;15(1):e0226557. doi: 10.1371/journal.pone.0226557 (PMC6988972; doi:10.1371/journal.pone.0226557)
Supplement: S7 Fig — (PDF) [file pone.0226557.s021.pdf]

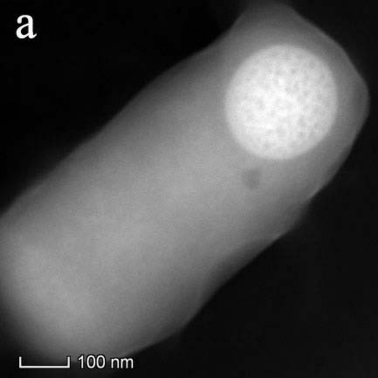
a
100 nm

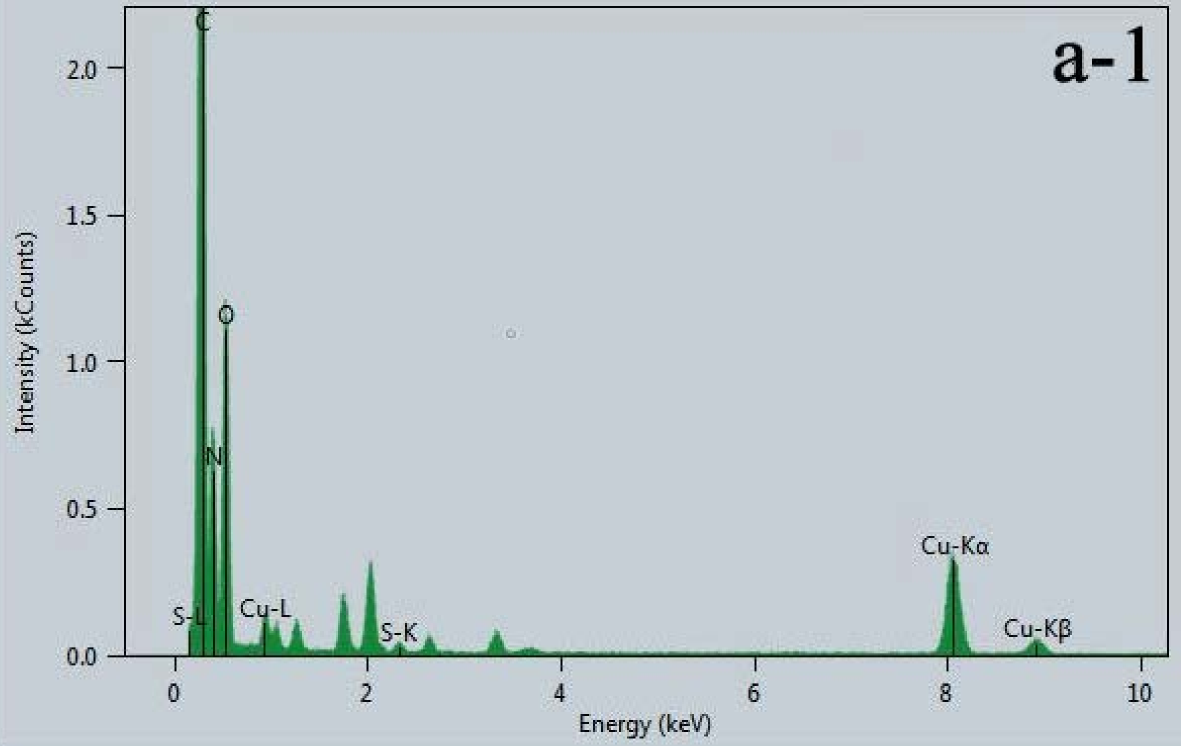
a-1
C
O
N
S-L
Cu-L
S-K
Cu-Kα
Cu-Kβ
Intensity (kCounts)
2.0
1.5
1.0
0.5
0.0
0
2
4
6
8
10
Energy (keV)

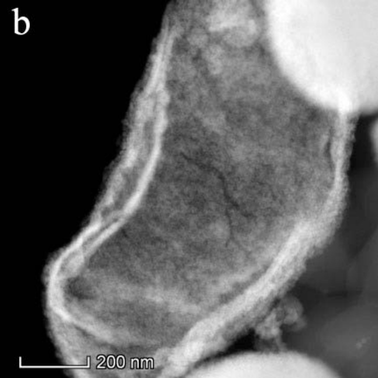

b
200 nm

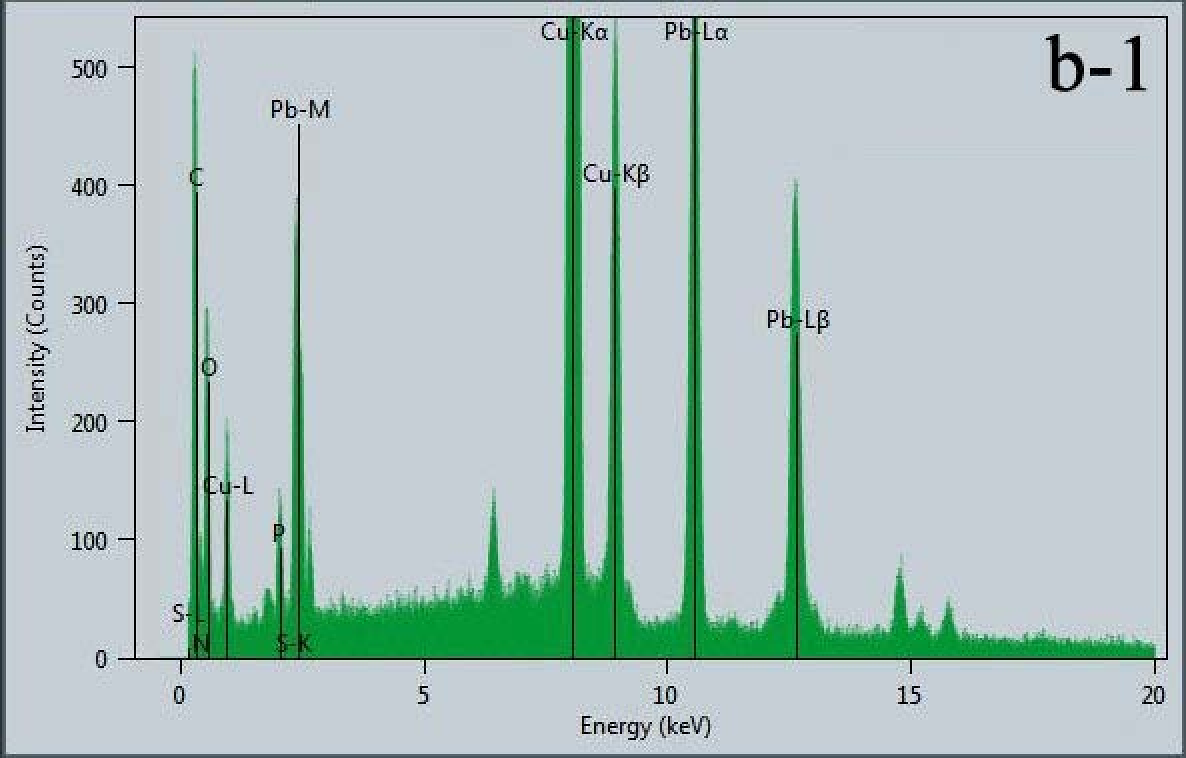
b-1
Intensity (Counts)
Energy (keV)
Cu-Kα
Pb-Lα
Pb-M
C
Cu-Kβ
Pb-Lβ
O
Cu-L
P
S-L
N
S-K
0
100
200
300
400
500
0
5
10
15
20

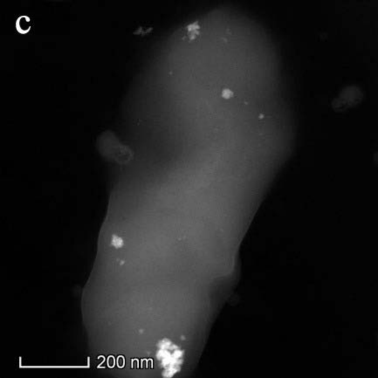
c
200 nm

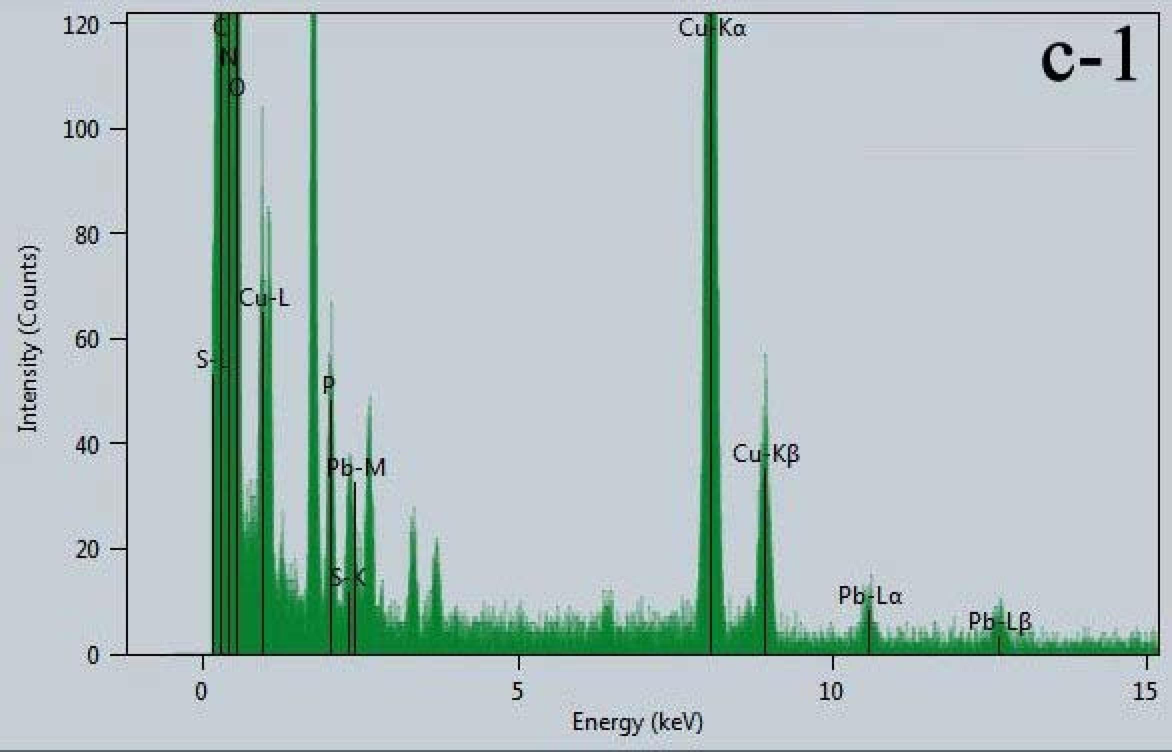
c-1
C
N
O
Cu-L
S-L
P
Pb-M
S-K
Cu-Kα
Cu-Kβ
Pb-Lα
Pb-Lβ
Intensity (Counts)
Energy (keV)
0
20
40
60
80
100
120
0
5
10
15
