# Supplementary material for: Pb2+ biosorption from aqueous solutions by live and dead biosorbents of the hydrocarbon-degrading strain Rhodococcus sp. HX-2
Source: PLoS One. 2020 Jan 29;15(1):e0226557. doi: 10.1371/journal.pone.0226557 (PMC6988972; doi:10.1371/journal.pone.0226557)
Supplement: S1 Equation — (PDF) [file pone.0226557.s022.pdf]

**Pseudo-first-order equation**

Pseudo-first-order equation has been widely used for analyzing the adsorption of an adsorbate from an aqueous solution following Eq (1):

$$\log(q_e - q_t) = \log q_e - \frac{tk_1}{2.303} \quad (1)$$

Where $q_e$ and $q_t$ are the amounts of heavy metal ions adsorbed (mg $g^{-1}$) at equilibrium and at time $t$ (min), respectively, and $k_1$ ($min^{-1}$) is the rate constant adsorption. Values of $k_1$ are calculated from the plots of log ($q_e$-$q_t$) versus t for the live and dead biosorbents.
