# Supplementary material for: Pb2+ biosorption from aqueous solutions by live and dead biosorbents of the hydrocarbon-degrading strain Rhodococcus sp. HX-2
Source: PLoS One. 2020 Jan 29;15(1):e0226557. doi: 10.1371/journal.pone.0226557 (PMC6988972; doi:10.1371/journal.pone.0226557)
Supplement: Attachment — Submitted filename: Response to Reviewer 1.docx [file pone.0226557.s023.docx]

List of Responses

Dear Editors and Reviewers:

Thank you for your letter and for the reviewers’ comments concerning our manuscript entitled “Biosorption of Pb^2+^ from aqueous solution by live and dead biosorbents of hydrocarbon-degrading strain *Rhodococcus* HX-2” (ID: PONE-D-19-26926). Those comments are all valuable and very helpful for revising and improving our paper, as well as the important guiding significance to our researches. We have studied comments carefully and have made correction which we hope meet with approval. Revised portion are marked in yellow in the paper. The main corrections in the paper and the responds to the reviewer’s comments are as flowing:

Responds to the reviewer’s comments:

Reviewer #1:

**1. The** **recycling experiments for the biosorption of Pb should be supplemented to ensure the stability.**

It is really true as Reviewer suggested that recycling experiments for the biosorption of Pb^2+^is essential.So, we decided to add the following to the article:

Recycling of biosorbent

In order to regenerate the biosorbent, Pb^2+^ loaded biosorbent (both live and dead) was treated with pH=1 HCl at 25 °C with a shaking speed of 200 rpm for 12 h. At the end of desorption, the biosorbent was collected by centrifugation at 11,000 *×g* for 10min, and the concentration of Pb^2+^ was determined as described above (ICP-OES). The regenerated biosorbent were re-used for further biosorption and the biosorption-desorption was repeated for five cycles.

S5 Fig.The recycling experiments (b)

The biosorption-desorption experiments was conducted by five cycles (as shown in S5a Figure). Compared with the first cycle, the biosorption capacity of bacteria by HCl elute at the fifth cycle was decreased from 114.2 to 79.9 mg/g (dead biosorbents) and from 62.6 to 38.8 mg/g (live biosorbents), respectively. The recycling efficiency was 70% and 61.9% for dead and live biosorbents. After five recycling experiments, the live and dead biosorbents still has a strong biosorption capacity, indicating that it can be applied to the actual metal solution biosorption.

**2. Does Pb harm bacterial colonies?**

We applied a dilution of HX-2 in the logarithmic growth phase to the plate, and each set of experiments was repeated three times. The results are as follows:

As can be seen in Figure 1, by counting the number of colonies by dilution coating, it can be seen that Pb^2+^ does not affect the growth of bacteria on LB solid medium (Figure 2). However, the number of colonies on LB plates with low concentration of Pb^2+^ is more than the control, which also proves that our previous conclusion that a small amount of Pb^2+^ promotes the growth of bacteria (manuscript Figure 1). As can be seen from Figure 3, Pb^2+^ did not change the surface morphology of the bacteria, and EDX did not show that the cells loaded Pb^2+^. This proves that a small amount of lead ions does not harm the bacterial colonies.

**Fig. 1.**Bacterial colonies in LB solid medium

(A: 0 mg/L Pb^2+^;B: 50 mg/L Pb^2+^;C: 100 mg/L; Pb^2+^; D: 200 mg/L Pb^2+^)

**Fig. 2.** Colony-Forming Units (CFU) of HX-2 in LB solid medium with different concentration of Pb^2+^

**Fig. 3.** HX-2 (SEM)in LB solid medium with different concentration of Pb^2+^ (a: 0 mg/L Pb^2+^;b: 50 mg/L Pb^2+^;c: 100 mg/L; Pb^2+^; d: 200 mg/L Pb2+)

**3. The whole expression of the manuscript should be carefully modified to be scientific and vigorous.**

We have made correction according to the Reviewer’s comments.

**4. A large body of literatures on related research works in toxic metal adsorption are missed.**

**Journal of Hazardous Materials, 2019, DOI: 10.1016/j.jhazmat.2019.120871. ACSNew J. Chem., 2019,43, 14575-14583.Applied Materials and Interfaces, 2019,11, 18165-18177. Journal of Nanomaterials, 2017, 2017, 7029731.**

As Reviewer suggested that we have not only added the above-mentioned literature, but also added many articles on the adsorption of heavy metals by live and dead adsorbents to enrich the content of our article.

We tried our best to improve the manuscript and made some changes in the manuscript. These changes will not influence the content and framework of the paper. We appreciate for Editors/Reviewers’ warm work earnestly, and hope that the correction will meet with approval. Once again, thank you very much for your comments and suggestions.
